# Supplementary figures and images for: Biocompatibility and Mechanical Properties of Carboxymethyl Chitosan Hydrogels
Source: Polymers (Basel). 2022 Dec 28;15(1):144. doi: 10.3390/polym15010144 (PMC9823898; doi:10.3390/polym15010144)

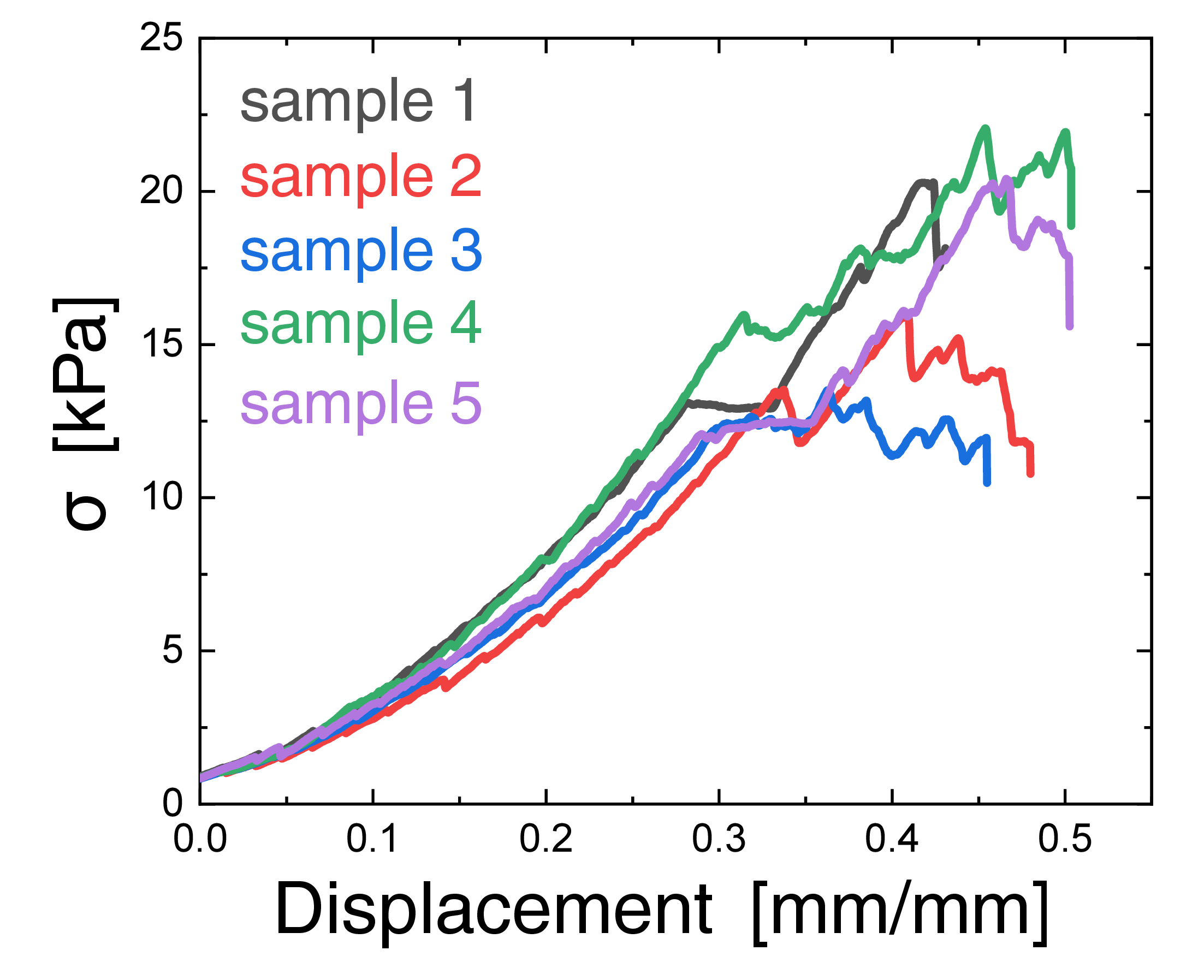

Supplement: Supplementary file 1 [file polymers-15-00144-s001.zip › FigureS1.tiff]

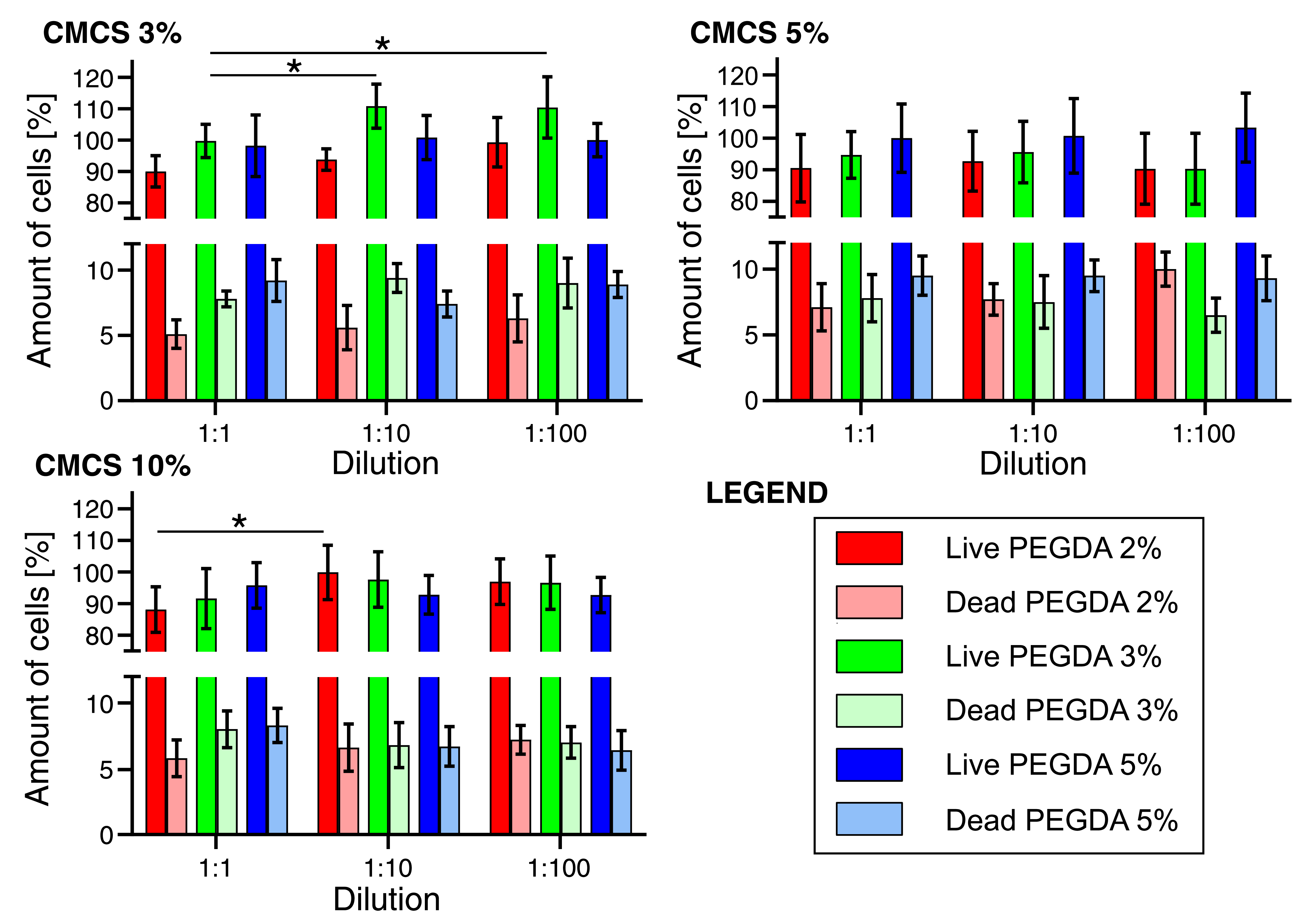

Supplement: Supplementary file 1 [file polymers-15-00144-s001.zip › FigureS2.tiff]
